# Prevalence and Associated Factors of Antenatal Depression among Women Attending Antenatal Care Service at Gondar University Hospital, Northwest Ethiopia

**DOI:** 10.1371/journal.pone.0155125

**Published:** 2016-05-06

**Authors:** Tadesse Awoke Ayele, Telake Azale, Kassahun Alemu, Zewditu Abdissa, Haregewoin Mulat, Abel Fekadu

**Affiliations:** 1 University of Gondar, College of Medicine and Health Sciences, Department of Epidemiology and Biostatistics, Gondar, Ethiopia; 2 University of Gondar, College of Medicine and Health Sciences, Department of Reproductive health, Gondar, Ethiopia; 3 University of Gondar, College of Medicine and Health Sciences, Department of Anesthesia, Gondar, Ethiopia; 4 University of Gondar, College of Medicine and Health Sciences, Department of Psychiatry, Gondar, Ethiopia; University of Oxford, UNITED KINGDOM

## Abstract

**Background:**

Depression is the most prevalent psychiatric disorder during pregnancy and is associated with psychosocial and clinical obstetric factors. Depressive disorders are not only common and chronic among women throughout the world but also principal sources of disability. The scarce information and limited attention to the problem might aggravate the consequence of the problem and can limit the intervention to be taken. Therefore, the current study was conducted to determine the prevalence and identify associated factors for antenatal depression.

**Methods:**

Institutional based cross-sectional study was conducted by taking a sample of 388 pregnant women coming for ANC service at Gondar University Hospital. Systematic random sampling technique was employed to recruit the study participants. Structured, pretested and interview administered questionnaire was used to collect related information while Beck Depression Inventory (BDI) was used to assess individuals`depression condition. A cut off point with high sensitivity and specificity was determined and internal consistency of the tool was checked (Cronbach alpha = 0.82). Ep Info V. 2002 and STATA 12 were used for data entry and analyses, respectively. Adjusted Odds Ratio with its 95% CI was used to declare the statistical significance of the factors.

**Results:**

Depression among pregnant women was found to be 23% (95%CI: 18.48%, 26.86%). Factors significantly associated with depression were: woman`s age (20 to 29, AOR = 0.18,95% CI:0.07,0.49), occupation (housewife, AOR = 2.57,95%CI:1.21,5.46, merchant and daily laborers, AOR = 3.44 (1.38,8.58), previous pregnancy (No, AOR = 4.74,95% CI:1.58,14.17) and previous ANC follow up pattern (irregular, AOR = 11.43,95% CI:3.68,35.49), no follow up, AOR = 11.98, 95% CI:4.73,30.33).

**Conclusion:**

Depression symptoms are common in pregnant mothers in the study area and interventions that would address the aforementioned factors would benefit to tackle further complications.

## Background

Depression is the fourth leading cause of disease burden and the largest cause of non-fatal burden, accounting for almost 12% of all total years lived with disability worldwide [1]. It is one of the most ancient and common diseases of the human race. Depressed patients are at least as heavily disabled as patients affected by other chronic diseases such as hypertension, rheumatoid arthritis and diabetes [2].

Depression is also the most prevalent psychiatric disorder during pregnancy. Almost one woman in four will experience depression at some point in her life, most commonly during the childbearing years [3]. Depressive disorders are not only common and chronic among women throughout the world but also principal sources of disability [1–3].

Antenatal depression often precedes postnatal depression [4] and causes great suffering to the woman and her family [5]. On top of that, untreated depression is associated with higher rates of mortality and morbidity. Similarly, discontinuation of antidepressant drug therapy in women with medication-responsive illness carries a high risk for relapse and suicide attempts [6]. More worryingly, perinatal depression has been found to be linked with infant undernutrition in many low-income countries [7].

Negativity and low caregiver responsiveness may contribute to high rates of insecure attachment found among infants of depressed mothers [8]. Children of depressed mothers are also at risk for slower cognitive development [9], low activity, difficulty interacting with unfamiliar adults, and unresponsiveness [10]. Biomedical consequences include an increased risk for breastfeeding problems [11], eating and sleep disturbances [12], and a reduced likelihood of receiving preventative health care[13] or daily vitamin supplementation [14]. Depressed mood during pregnancy has also been associated with poor attendance at antenatal clinics, substance misuse, low birth weight, and preterm delivery [7,8].

Epidemiological data suggest roughly 15% of women are depressed at any one time [15], and some research suggests that mothers of young children may have rates of depression even higher than the general population of women [16]. The prevalence of maternal depression in low- and middle- income countries is estimated at 15%–28% in Africa and Asia [9–12], 28%–57% in Pakistan [13], and 35%–50% in Latin America [14].

A cohort study of women attending a district hospital antenatal clinic in Goa, and community-based study from Tamil Nadu, India, reported a prevalence of 23% and 19.8%, respectively [17,18]. Another community cohort study from Pakistan reported a prevalence of 28% [19]. A Meta-analysis shows the prevalence of depression during pregnancy is also high, ranging from 0.5% to 51% [11]. In Ethiopia, very few published studies are there concerning antenatal depression. Those published papers show the prevalence of depression among pregnant women ranging from 4.4% to 12% [20–22].

Different studies conducted so far in different area reported risk factors for depression among pregnant mothers: young age [12], low income [13], lower educational attainment [12], history of depression [12,14], a history of miscarriage and pregnancy termination [8], and a history of childhood sexual abuse [20], concomitant high anxiety in pregnancy [23], low self-esteem [19] and low social support [24,25].

Depression in pregnancy may diminish one's capacity for self-care, including inadequate nutrition, drug or alcohol abuse, and poor antenatal clinic attendance, all of which may compromise a woman's physical and mental health and may reduce optimal fetal monitoring or restrict the growth and development of the fetus. Although nearly 90% of the worlds’ children live in low- and middle-income countries, we know little about the prevalence of maternal depression in Ethiopia. Therefore, the current study was conducted to determine the magnitude of depression and its associated factors among pregnant mothers attending antenatal care clinic at Gondar University teaching hospital.

## Methods

### Study setting and design

A depression screening questionnaire was administered on a number of mothers visiting ANC clinic on a single point time from October 2011 to January 2012 to estimate the prevalence of depression and its associated factors among pregnant mothers at Gondar University teaching hospital. Gondar is located about 723 km from Addis Ababa and 65 km North from Lake Tana where the Blue Nile starts. Gondar University Hospital provides tertiary care to the population of Gondar and its neighboring states, and most of its patients come from the lower socioeconomic strata. The Hospital has an antenatal clinic, which gives antenatal care services every day. Annually about 17,280 pregnant mothers visited the hospital for ANC services including first, second, third and fourth visits.

### Sample size determination and procedure

All pregnant women attending antenatal care service clinic were taken as a source population while those pregnant mothers who were available during data collection period were considered as a study population. As per the information obtained from the antenatal care service, about 20 pregnant women used to visit the hospital for antenatal care service daily. The total number of pregnant mothers who will visit the clinic within the three months of data collection period was calculated and used to determine the interval. Therefore, systematic random sampling technique was used to include every three pregnant mothers available at the clinic during the data collection period. A single population proportion formula with an assumption of 95% confidence level, a 4% margin of error and a 21% prevalence [26] of depression among pregnant mothers and a non-response rate of 5% was taken to determine a final sample size of 418.

### Data collection method and instrument

Data were collected using structured and pretested questionnaire by face to face interview technique. Trained nurses working in the ANC clinic collected the data. The questionnaire enclosed socio-demographic data, Beck Depression Inventory (BDI) and structured questions for assessment of associated factors. BDI was translated into Amharic and back to English by two different languages and psychiatrist for checking its consistency. BDI was developed and revised to reflect the revision in the current DSM-IV-TR. BDI had been extensively tested for content validity.

Beck Depression Inventory was composed of 21 questions, each with four possible responses. Each response was assigned a score ranging from zero to three, indicating the severity of a symptom. The overall value of a scale ranges from zero to 63 [27]. A cut of point with high sensitivity and specificity was determined by undertaking ROC analysis and calculating the area under the curve in STATA 12. Therefore, a pregnant mother with a score of 15 and less was considered as normal while a mother with BDI score of 16 and more was considered as depressed. The internal validity of the instrument was also checked (Cronbach alfa = 0.826) which ensures the internal consistency of the instrument.

### Data quality assurance and analysis

Data was entered to Ep Info version 2002 after checking for completeness and imported to STATA 12 for further analysis. Exploratory and statistical data analysis was done and results were presented using tables. Bivariate logistic regression was first fitted to identify potential confounding factors and variables with a p-value less than 0.2 were entered to multiple logistic regression models using backward selection method to identify associated factors with depression. Adjusted odds ratio with its 95% confidence interval was calculated to report the strength and significance of the association.

### Ethical consideration

Ethical approval was obtained from Institutional Review Board (IRB) of research and community service of the University of Gondar, college of medicine and health science. Permission letter from Gondar university hospital was also secured before data collection. Pregnant mothers were asked and written consent was obtained after discussing the confidentiality, the purpose, benefit and possible risks of the study.

## Results

### Socio-demographic characteristics of the pregnant mothers

A total of 388 pregnant women with gestational age ranging between 16 to 39 weeks were included with a response rate of 92.82%. The median BDI score was found to be 11.50 (IQR: 10.00–15.00). The median age of the women was 25 years with an interquartile range of (28.75–22.00) years. Nearly one in three women educated until they reach high school and most 350 (90.21%) of them were married. Nearly two-in-five (41.49%) of the women were a housewife and most (83.25%) were from the urban area. Nearly one in three women (36%) earns less than 50 dollars per month (Table 1).

**Table 1:** Sociodemographic characteristics of pregnant women who attained antenatal care services at Gondar University Hospital, October 2011 to January 2012.

Variable	Category	Number	%
age	14–19	26	6.70
	20–29	274	70.62
	>30	88	22.68
Education status	illiterate	71	18.30
	elementary	69	17.78
	high School	143	36.86
	Diploma & above	105	27.06
Marital status	Single	19	4.90
	Married	350	90.21
	Divorced	11	2.84
	Separated	5	1.29
	widowed	3	0.77
Occupation	Gov't employee	100	25.77
	NonGov't employee	58	14.95
	Housewife	161	41.49
	Other	69	17.78
Resident	Rural	65	16.75
	Urban	323	83.25
Income(in birr)	<1000	140	36.08
	1001–2000	131	33.76
	2000–3000	62	15.98
	>3000	55	14.18

### Obstetric history of pregnant mothers

For 158 (40.72%) of the women, the current pregnancy was their first pregnancy. Only 28 (7.22%) had four or more number of gravidity. Of 262 pregnant women who had at least one history of pregnancy, most 171 (65.27%) had experienced previous pregnancy complication. Whereas, 172 (44.33%) had experienced a complication during labor. About one in five women experienced abortion in their previous pregnancy in which near half (47.44%) were induced (Table 2).

**Table 2:** Obstetric history of pregnant women who attained ANC at Gondar University Hospital, October 2011 to January 2012.

Variable	Category	Number	%
**Number of previous Pregnancies (gravidity)**			
	No	158	40.72
	One	113	29.12
	Two	56	14.43
	Three	33	8.51
	Four & above	28	7.22
**History of previous pregnancy complication**			
	Yes	171	65.27
	No	91	34.73
**History of complication in previous labor**			
	Yes	172	44.33
	No	216	55.67
**Pattern of antenatal care in the previous pregnancy**			
	Regular	168	43.30
	Irregular	63	16.24
	Not at all	157	40.46
**Number of previous abortion**			
	One	60	77.92
	Two	15	19.48
	Three & Above	2	2.60
**Pattern of previous abortion**			
	Spontaneous	41	52.56
	Induced	37	47.44
**Pattern of current pregnancy**			
	Planned	350	90.21
	unplanned	38	9.79
**Number of live birth**	No	158	40.72
	One	114	29.38
	Two	59	15.21

### Psychosocial history of the pregnant mothers

Most of the women 566 (94.33%) had very good support from their husband and equivalent proportion were satisfied. Moreover, most of the husbands 239 (61.6%) had very good feeling about the pregnancy. Nearly two in three 276 (76.29%) women reported that the support of the community was good. Despite the fact that most 351 (90.46%) of the women were satisfied with the antenatal care services, 30 (7.73%) women were abused by the health professionals working in the clinic. One hundred twenty-one (31.19%) of them revealed as antenatal care has a solution for the psychological problems (Table 3).

**Table 3:** Psychological history of pregnant women who visited Gondar University Hospital, October 2011 to January 2012.

Variable	Category	Number	%
Husband Feeling	Very Good	239	61.60
	Good	135	34.79
	No difference	14	3.61
Husband Support	Very Good	366	94.33
	Good	13	3.35
	Not Good	9	2.32
Community Support	Very Good	89	22.94
	Good	296	76.29
	Not Good	3	0.77
Satisfaction by the husband support			
	Yes	367	94.59
	No	21	5.41
ANC visit	very helpful	310	79.90
	may be	72	18.56
	Only for fetus	6	1.55
Satisfaction by ANC	Yes	351	90.46
	No	37	9.54
Solution for psychological problem			
	Yes	121	31.19
	No	267	68.81
Abuse by health professional			
	Yes	30	7.73
	No	358	92.27

### Prevalence of Depression in pregnant mothers

**Fig 1:**
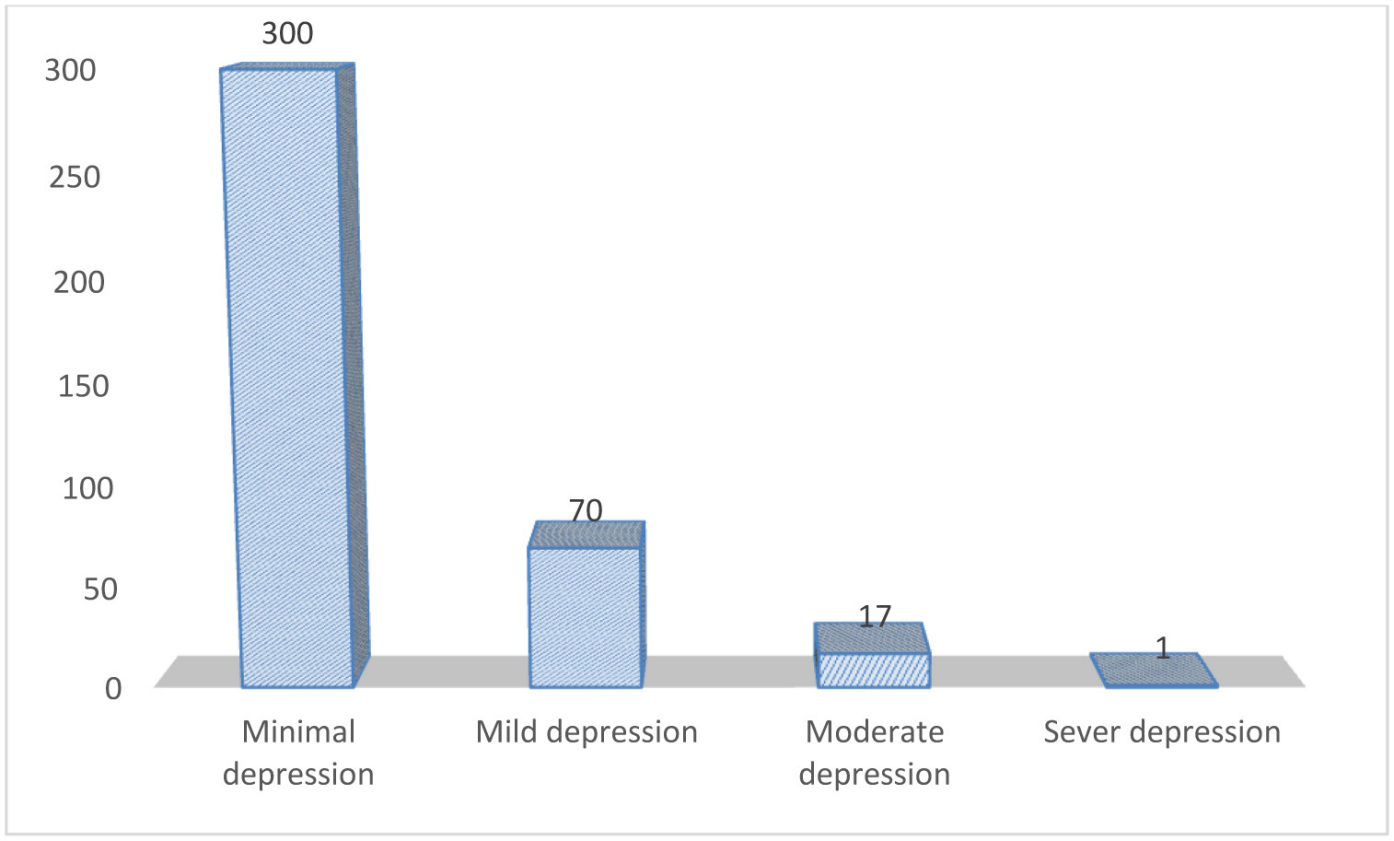
Bar chart showing the distribution BDI score for pregnant women attaining antenatal care service at Gondar University Hospital ANC clinic, October 2011 to January 2012.

The 21-items of the Beck Depression Inventory (BDI) were summed and single variable was generated. The new variable ranges from 0 to 63 in absolute value. A total of 70 (18%) women had mild depression (BDI score of 16 to 30) and 17 (4.38%) of pregnant women had moderate depression (BDI score of 31 to 46). Whereas, most (77.32%) pregnant women had BDI score 0 to 15 which indicates minimal depression. Only one mother found to have severe depression (BDI score greater than 46) (Fig 1).

**Fig 2:**
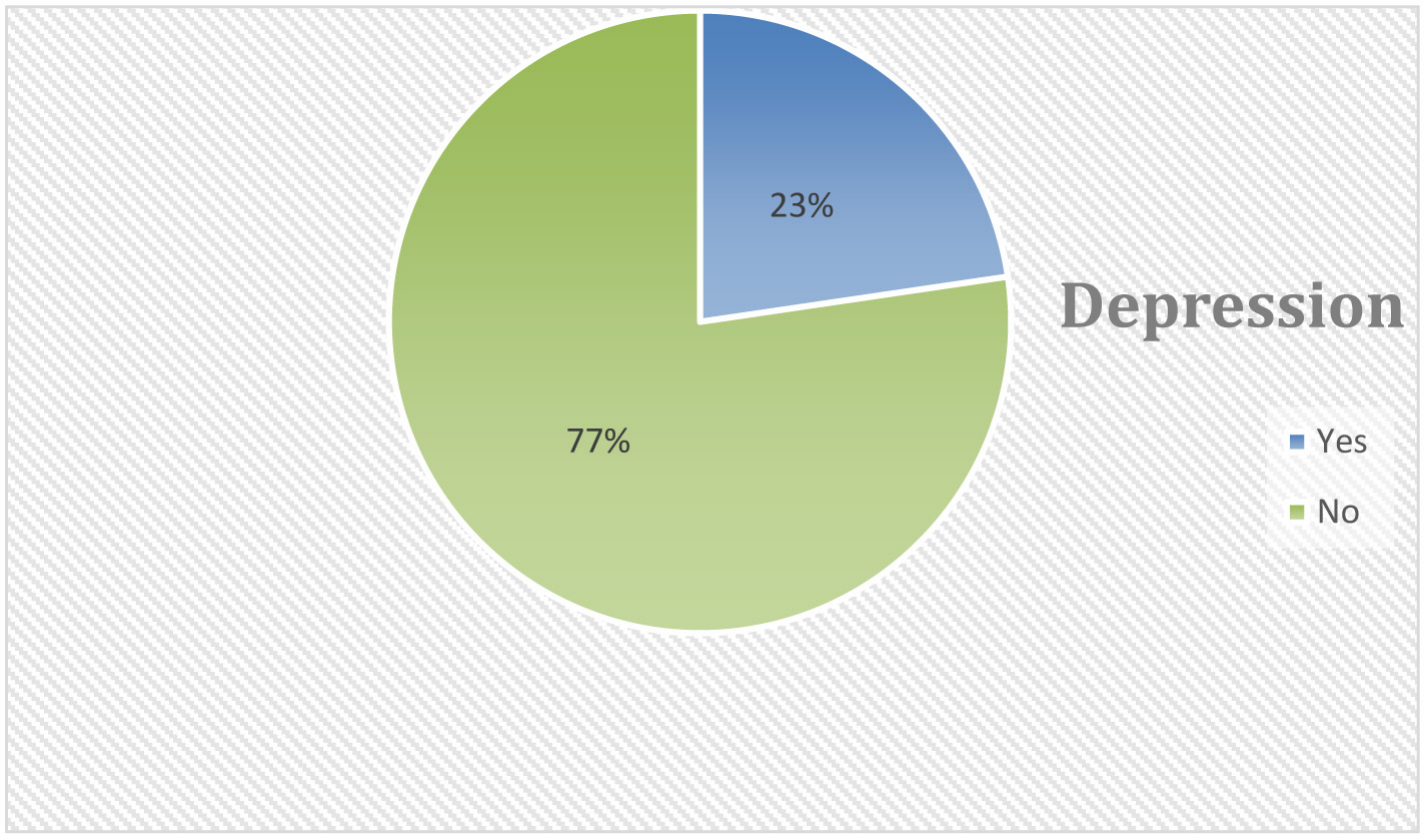
Pie chart for the distribution of depression among pregnant women at Gondar university hospital ANC clinic, October 2011 to January 2012.

We further categorize the depression score into two levels (depressed and not depressed). Scores greater than or equal to 16 were used as an indicator for the existence of depression and no depression otherwise. As a result, nearly one in four (23%, 95%CI; 18.49%, 26.86%) women had an indication of depression related to pregnancy (Fig 2).

### Factors associated with Depression

Socio-demographic factors, obstetric factors, and psychosocial factors were used to identify statistically significant factors. Among all covariates, age group, occupation, number of previous pregnancy, previous ANC pattern, previous pregnancy complication, type of current pregnancy, satisfaction by ANC services, joy from the pregnancy and solution for psychological problem by the antenatal care services were found to have p-value less than 0.2 from bi-variable logistic regression and considered for the multiple logistic regression model. The model goodness of fit was tested using Hosmer and Lemeshow test and the p-value was found to be 0.635, which revealed as the model is good.

Pregnancy between 20 to 29 years of age had reduced the risk of depression as compared to age less than 20 years. The risk of depression for women whose age ranges between 20 to 29 years reduced by 0.184 (95%CI: 0.07, 0.50) times as compared to women whose age ranges between 14 to 19 years. The odds of depression increased by 2.57 times (95%CI: 1.21, 5.46) for housewife’s than a government employee. History of previous pregnancy has reduced the risk of depression. Those pregnant women who did not have the previous history of pregnancy were about five times (95%CI: 1.59, 14.17) more at risk of depression than women who had at least two number of previous pregnancy (Table 4).

**Table 4:** Factors associated with depression among pregnant women who attain antenatal care service at Gondar University Hospital, October 2011 to January 2012.

		Depression			
Variable	Category	No	Yes	COR(95%CI)	AOR(95%CI)
Age group	14–19	12	14	1	
	20–29	223	51	0.19(0.08; 0.45)	**0.18(0.07; 0.49)**
	>30	65	23	0.30(0.12; 0.75)	0.33(0.11; 1.00)
Occupation	Gov. employee	87	13	1	
	NG employee	46	12	1.75(0.74; 4.13)	2.35(0.93; 5.95)
	House wife	118	43	2.44(1.24; 4.81)	**2.57(1.21; 5.46)**
	Other*	49	20	2.73(1.25; 5.96)	**3.44(1.38; 8.58)**
Previous pregnancy	None	120	38	1.16(0.65: 2.07)	**4.74(1.58;14.17)**
	One	88	25	1.04(0.55: 1.95)	1.19(0.57; 2.46)
	Two & above	92	25	1	1
Previous ANC pattern	Regular	127	41	1	1
	Irregular	36	27	2.21(1.23; 3.97)	**11.43(3.68;35.49)**
	None	137	20	5.14(2.59; 10.19)	**11.98(4.73; 30.33)**
Previous pregnancy complication	Yes	135	36	1	
	No	54	37	2.57(1.47; 4.48)	-
Current pregnancy	Planned	273	77	1	
	unplanned	27	11	1.44(0.68; 3.04)	-
Satisfaction by ANC	Yes	273	78		1
	No	27	10	3.37(1.38; 8.22)	4.78(1.74:13.11)

*Others = merchant and daily laborers.

The history of antenatal care service pattern was associated with depression of pregnant women. The odds of depression increased by 12 times (95%CI: 4.74, 30.33) for women who did not have a history of antenatal care service as compared to women who had regular visit history. Visiting antenatal care service irregularly has also increased the risk of depression. The odds of depression increased by 11 times (95%CI: 3.68, 35.50) for women who had an irregular visit as compared to regular visits. Satisfaction of women by the antenatal care service provision has reduced the risk of depression. The odds of depression increased by 4.78 times (95%CI: 1.74,13.11) for women who were not satisfied by the service provision (Table 4).

## Discussion

The prevalence of depression among antenatal care attendant mothers in the current study was found to be 23% (95%CI: 18.5%, 26.9%). The current estimate is by far higher than the three prospective cohort studies conducted at the different time in Butajira DSS (12%, 9.2% and 7.3%) [20–22], the two studies from Australia (16.9%, 8.9%) [28,29] and study from southwestern Finland (7.7%) [30]. This might be because of the difference in study design, study setting, study year, socio-demographic and type of screening tool used. The current study was institution based while the previous three studies were community-based, the current studies used Beck Depression Inventory scale while the previous studies used self-reported questioner (SRQ-20). Even though the three studies from Australia and Finland were the institutionally based cross-sectional studies that might make it similar with the current study, the tool used, year of study and the socio-demographic difference might account for the difference.

The estimate of the current study is consistent with studies reported from South Africa [17], Jordan [31], India [18], a systematic review conducted in Africa [32] and a cross-sectional study from Southwestern Ethiopia [33]. In the other hand, the estimate of the current study is lower than a two studies conducted in Turkey [34,35], Tanzania [36] and Jamaica [13]. The tool used for measuring depression and cutoff point used to categorize the mother's depression condition is completely different from the current study. On top of that, the study time, setting and stage of pregnancy at which the current data was collected could be considered as a source of variation in estimated between the current and compared studies.

The current study was also identified different factors that had an association with depression during pregnancy. Age of the pregnant mother was one of the factors that had a significant association with depression. The risk of depression for women whose age ranges between 20 to 29 years was 82% lower as compared to mother’s age between 14 to 19 years, which is a contradictory finding with the two studies conducted in Turkish [35,37], a cross-sectional study from China [38], and a prospective study from Butajira DSS site [20]. However, our study result is consistent with an institution based cross-sectional study from eastern Iowa [39] which published a negative correlation between mothers age and depression scale. All of the studies that had contradictory findings with the current study were community-based studies, socio-demographic difference, the variation in measurement tool and cut off point used could account for the difference.

The other factor that had a significant association with maternal depression was their occupation. The odd of depression was 2.57 times higher among housewives as compared to government employees. This is consistent with a study conducted among Swedish mothers [40]. Similarly, a systematic review conducted so far among low and lower middle-income countries reported a protective effect of having a permanent job for antenatal depression [41]. This might support the mothers to have an intimate friend and to share information concerning issues with her pregnancy. The other factor, which increased the odd of depression, was gravidity. Prim gravidas were five times at higher risk of being depressed as compared to a mother with at least two number of previous pregnancy. This is supported with other similar study conducted so far [20,35,39]. This might be because of the fact that a first pregnancy for a mother will pose different psychosocial problems and fear of complications during the course of the pregnancy.

Alike studies so far [31,36,42], in the current study mother who failed to book their ANC and made irregular visit were 12 and 11 times at higher risk for depression. Similarly, mothers who were not satisfied by the ANC service were nearly five times at higher risk for depression. This might happen because mothers who had at least one ANC and made regular visit would get proper counseling on different issues of the pregnancy course that will help them to build their self-esteem. Even though, the mothers self-steem was not assessed in the current study, similar studies so far reported [9,20,31], its protective effect for depression. On top of that being satisfied by the ANC service provided would motivate the mothers to made regular visit so that they could get continued support from the health professional.

Unlike other studies, the current study did not found any association between previous pregnancy complication and outcome, income, residence, pregnancy condition (being wanted or unwanted), intimate partner support, and substance use. However, the study identified factors related to the specific study area that can help to take appropriate intervention. Our study has some limitations. The major limitation of this study was the use of the BDI which could overestimate the prevalence of depression through the somatic symptoms which can exist normally during pregnancy. Moreover, the instrument has not been locally validated although it has been used in previous studies of women with pelvic floor diseases [43]. The study was institution based which could limit its generalizability, using questionnaire for measuring depression could also limit its generalizability to clinically identified cases, fail to appropriately measure factors like social support would introduce residual confounding to the study result and cross-sectional nature of the study would also limit its ability to establish a temporal relationship. Therefore, the interpretation and usage of the study should consider these limitations.

## Conclusion and Recommendation

The prevalence of antenatal depression in this population was relatively higher than studies reported in Ethiopia and from a review of other African countries. Younger age and being government employ were protective for depression while primigravida, making an irregular visit to not attending ANC service and not satisfied with ANC serves were the factors that increased the odds of depression. Taking targeted intervention and early screening of pregnant mothers for depression is crucial in order to prevent its further consequences.

## References

[1] Patel V, Rodrigues M, DeSouza N (2002) Gender, poverty, and postnatal depression: a study of mothers in Goa, India. Am J Psychiatry: 159:143–157. 11772688 10.1176/appi.ajp.159.1.43

[2] Organization.. WH (2010) The world health report. Geneva: WHO.

[3] Rahman A, Iqbal Z, Bunn J (2004) Impact of maternal depression on infant nutritional status and illness: a cohort study. Arch Gen Psychiatry 61:946–952. 15351773 10.1001/archpsyc.61.9.946

[4] Black M, Baqui A, Zaman K, McNary S, Arifeeen S (2007) Depressive symptoms among rural Bangladeshi mothers: Implications for infant development. Journal of Child Psychology and Psychiatry 48: 764–772. 17683448 10.1111/j.1469-7610.2007.01752.x

[5] Rahman A, Iqbal Z, Bunn J, Lovel H, Harrington R (2004); Impact of maternal depression on infant nutritional status and illness: a cohort study. Arch Gen Psychiatry 61: 946–952. 15351773 10.1001/archpsyc.61.9.946

[6] Rahman A, Iqbal Z, Harrington R (2003) Life events, social support, depression and childbirth: perspectives from a rural population in a developing country. Psychol Med 33: 1161–1167. 14580070 10.1017/s0033291703008286

[7] Patel V, Rahman A, Jacob KS, Hughes M (2004) Effect of maternal mental health on infant growth in low income countries: new evidence from South Asia. BMJ 328: 820–823. 15070641 10.1136/bmj.328.7443.820 PMC383383

[8] Rahman A, Bunn J, Lovel H, Creed F (2007) Maternal depression increases infant risk of diarrheal illness: A cohort study. Arch Dis Child 92: 24–28. 16966339 10.1136/adc.2005.086579 PMC2083137

[9] Leigh B, Milgrom J (2008) Risk factors for antenatal depression. BMC Psychiatry 8:24 10.1186/1471-244X-8-24 18412979 PMC2375874

[10] Qiu C, Sanchez SE, Lam N, Garcia P, Williams MA (2007) Associations of depression and depressive symptoms with preeclampsia: results from a Peruvian case-control study. BMC Women's Health 7:15 17900360 10.1186/1472-6874-7-15 PMC2148036

[11] Grote NK, Bridge JA, Gavin AR, Melville JL, Iyengar S, et al (2010) A meta analysis of depression during pregnancy and the risk of preterm birth, low birth weight, and intrauterine growth restriction. Arch Gen Psychiatry 67: 1012–1024. 10.1001/archgenpsychiatry.2010.111 20921117 PMC3025772

[12] Rahman A, Creed F (2007) Outcome of prenatal depression and risk factors associated with persistence in the first postnatal year: prospective study from Rawalpindi, Pakistan. Journal of Affective Disorders 100: 115–121 17098291 10.1016/j.jad.2006.10.004 PMC1894757

[13] Wissart J, Parshad O, Kulkarni S (2005) Prevalence of pre- and postpartum depression in Jamaican women. BMC Pregnancy and Childbirth 5: 15 16277665 10.1186/1471-2393-5-15 PMC1310611

[14] DaCosta D, Larouche J, Dritsa M, Brender W (2000) Psychosocial correlates of prepartum and postpartum depressed mood. Journal of Affective Disorders 59: 31–40. 10814768 10.1016/s0165-0327(99)00128-7

[15] Rochat T, Tomlinson M, Newell M, Stein A (2009) Depression among pregnant women testing for HIV in rural South Africa: Implications for VCT. 9th International AIDS Impact Conference; Botswana.

[16] DeBruin G, Swartz L, Tomlinson M, Cooper P, Molteno C (2004) The factor structure of the Edinburgh Postnatal Depression Scale in a South African peri-urban settlement. S Afr J Psychol 34: 113–121.

[17] Lawrie T, Hofmeyr G, de Jager M, Berk M (1998) Validation of the Edinburgh Postnatal Depression Scale on a cohort of South African women. S Afr Med J 88: 1340–1344. 9807193

[18] Dawson D, Grant B, Stinson F (2005) The AUDIT-C: screening for alcohol use disorders and risk drinking in the presence of other psychiatric disorders. Compr Psychiatry 46: 405–416 16275207 10.1016/j.comppsych.2005.01.006

[19] (2009) Center on the Developing Child at Harvard University, Maternal Depression Can Undermine the Development of Young Children. Working Paper No. 8. http://www.developingchild.harvard.edu

[20] Hanlon C, Medhin G, Alem A, Tesfaye F, Lakew Z, et al (2009) Impact of antenatal common mental disorders upon perinatal outcomes in Ethiopia: the P-MaMiE population-based cohort study. Trop Med Int Health 14(2): 156–166. 10.1111/j.1365-3156.2008.02198.x 19187514

[21] Ross J, Hanlon C, Medhin G, Alem A, Tesfaye F, et al (2010) Perinatal mental distress and infant morbidity in Ethiopia: a cohort study. Arch Dis Child Fetal Neonatal Ed 2011 96(1). 10.1136/adc.2010.183327 PMC3009468 20667895

[22] Servili C, Medhin G, Hanlon C, Tomlinson M, Worku B, et al (2010) Maternal common mental disorders and infant development in Ethiopia, the P-MaMiE Birth Cohort. BMC Public Health 10:693 10.1186/1471-2458-10-693 21073710 PMC3091583

[23] Stewart DE, Robertson E DC-L, Grace S, Wallington T (2003) Postpartum Depression: Literature review of risk factors and interventions. Toronto. http://www.toronto.ca/health/pdf/ppd_e_chap1.pdf

[24] Cox J, Holden J, Sagovsky R (1987) Detection of postnatal depression. Development of the 10-item Edinburgh Postnatal Depression Scale. Br J Psychiatry 150: 782–786. 3651732 10.1192/bjp.150.6.782

[25] Murray D, Cox J (1990) Screening for depression during pregnancy with the Edinburgh depression scale (EPDS). J Reprod Infant Psychol 8: 99–107.

[26] Jebena mG, Taha M, Nakajima M, Lemieux A, Lemessa F, et al (2015) ousehold food insecurity and mental distress among pregnant women in Southwestern Ethiopia: a cross sectional study design. BMC Pregnancy and Childbirth 15:250 10.1186/s12884-015-0699-5 26449375 PMC4599660

[27] Beck A.T, Ward C.H, Mendelson M, Mock J, Erbaugh J (1961) An inventory for measuring depression. Archives of General Psychiatry 4: 561–571. 13688369 10.1001/archpsyc.1961.01710120031004

[28] Milgrom J, Gemmill AW, Bilszta JL, Hayes B, Barnett B, et al (2008) Antenatal risk factors for postnatal depression: A large prospective study Journal of Affective Disorders 108: 147–157. 18067974 10.1016/j.jad.2007.10.014

[29] Leigh B, Milgrom J (2008) Risk factors for antenatal depression, postnatal depression and parenting stress BMC Psychiatry 8:24 10.1186/1471-244X-8-24 18412979 PMC2375874

[30] Pajulo M, Savonlahti E, Sourander A, Helenius H, Piha J (2001) Antenatal depression, substance dependency and social support Journal of Affective Disorders 65 9–17. 11426516 10.1016/s0165-0327(00)00265-2

[31] Mohammad KI, Gamble J, Creedy DK (2011) Prevalence and factors associated with the development of antenatal and postnatal depression among Jordanian women Midwifery 27: e238–e245. 10.1016/j.midw.2010.10.008 21130548

[32] Sawyer A, Ayers S, Smith H (2010) Pre- and postnatal psychological wellbeing in Africa: a systematic review. J Affect Disord 123: 17–29. 10.1016/j.jad.2009.06.027 19635636

[33] Jebena MG, Taha M, Nakajima M, Lemieux A, Lemessa F, et al (2015) Household food insecurity and mental distress among pregnant women in Southwestern Ethiopia: a cross sectional study design. BMC Pregnancy and Childbirth 15:250 10.1186/s12884-015-0699-5 26449375 PMC4599660

[34] Aktas S, Calik KY (2015) Factors affecting Depression During Pregnancy and the Correlation Between Social Support and Pregnancy Depression. Iran Red Crescent Med J 17. 10.5812/ircmj.16640 PMC4601205 26473071

[35] Golbasi Z, Kelleci M, Kisacik G, Cetin A (2010) Prevalence and Correlates of Depression in Pregnancy Among Turkish Women Matern Child Health J 14: 485–491. 10.1007/s10995-009-0459-0 19238527

[36] Kaaya SF, Mbwambo JK, Kilonzo GP, Van Den Borne H, Leshabari MT, et al (2010) Socio-economic and partner relationship factors associated with antenatal depressive morbidity among pregnant women in Dar es Salaam, Tanzania. Tanzan J Health Res 12: 23–35. 20737826 10.4314/thrb.v12i1.56276

[37] Caliskan D, Oncu B, Kose K, Ocaktan ME, Psychosom J, et al (2007 12) Depression scores and associated factors in pregnant and non-pregnant women: a community-based study in Turkey. Obstet Gynaecol 28: 195–200. 10.1080/01674820701450649 17852661

[38] Zeng Y, Cui Y, Li J (2015) Prevalence and predictors of antenatal depressive symptoms among Chinese women in their third trimester: a cross-sectional survey. BMC Psychiatry: 15:66 10.1186/s12888-015-0452-7 25879965 PMC4387591

[39] Koleva H, Stuart S, O’Hara MW, Bowman J (2011) Risk factors for depressive symptoms during pregnancy Arch Womens Ment Health 14: 99–105. 10.1007/s00737-010-0184-0 20872153 PMC3433272

[40] Rubertssona C, WaldenstrÖma U, Wickbergb B, Rådestadc I (2005) Depressive mood in early pregnancy and postpartum: prevalence and women at risk in a national Swedish sample Journal of Reproductive and Infant Psychology 23.

[41] Fisher J, Mello MCd, Patel V, Rahman A, Tran T, et al (2012) Prevalence and determinants of common perinatal mental disorders in women in low- and lower-middle-income countries: a systematic review. Bull World Health Organ 90: 139–149G. 10.2471/BLT.11.091850 PMC3302553 22423165

[42] Marcus SM (2009) Depression during pregnancy: rates, risks and consequences—Motherisk Update 2008. Can J Clin Pharmacol 16: e15–22. 19164843

[43] Megabiaw B, Awoke T, Adefriss M, Azale T, Awoke A (2013) Depression among women with obstatric festula and pelvic organ prolapse in northwest Ethiopia BMC Psychiatry 13. 10.1186/1471-244X-13-236 PMC3849390 24070342

